# Trends in Female Authorship in Major Journals of 3 Oncology Disciplines, 2002-2018

**DOI:** 10.1001/jamanetworkopen.2021.2252

**Published:** 2021-04-06

**Authors:** Anirudh Yalamanchali, Emily S. Zhang, Reshma Jagsi

**Affiliations:** 1 Medicine Institute, Cleveland Clinic Foundation, Cleveland, Ohio; 2 Head and Neck Institute, Cleveland Clinic Foundation, Cleveland, Ohio; 3 Department of Radiation Oncology, University of Michigan, Ann Arbor

## Abstract

**Importance:**

Gender disparity exists among authors of the oncology literature.

**Objective:**

To quantify trends in authorship by gender within a comprehensive data set of Medline-indexed oncology articles in medical journals with high impact factors.

**Design, Setting, and Participants:**

This cross-sectional study used Medline citations to examine archives of research journals from 3 disciplines in the oncology literature. Authors from all oncology-related articles with Medical Subject Headings terms assigned from 2002 to 2018 from 13 general oncology/medicine, radiation oncology, and surgical journals were included for analysis, encompassing clinical trials, observational studies (excluding case reports), reviews, systematic reviews, meta-analyses, and all other published articles. Data were analyzed between April and May 2020.

**Exposures:**

Authors were assigned genders based on societal naming norms via third-party gender identification service Gender-API.com. This assignment was internally validated based on manually obtained publicly available data on the internet.

**Main Outcomes and Measures:**

Trend in female authorship over time while considering journal type, authorship position, and article type.

**Results:**

A total of 420 526 authors from 58 368 articles were found, of which 400 945 were assigned a gender based on their name. In total, 29.5% (95% CI, 29.4%-29.6%) of authors were identified as female, rising from 25.5% (95% CI, 24.7%-26.3%) in 2002 to 31.7% (95% CI, 31.2%-32.3%) in 2018. Each subgroup of primary article type by journal type saw a rise in female authorship over the studied period. For primary articles, last authors were less likely to be women than first authors, regardless of journal type, year, and primary article type (eg, general oncology: odds ratio [OR], 0.60; 95% CI, 0.52-0.69). For general oncology articles, women were less likely to be authors of clinical trials at each authorship position than authors at that respective position for observational studies (first, second, and last authors: OR, 0.58; 95% CI, 0.49-0.67; other authors: OR, 0.84; 95% CI, 0.79-0.89). This difference was not seen for radiation oncology or surgical oncology journals.

**Conclusions and Relevance:**

This cross-sectional study found that female authorship in oncology research literature has increased. However, there remains a dearth of female senior authors, and the overall rise in female authorship has not kept up with the rise in female oncology faculty.

## Key Points

**Question:**

How has the representation of women as authors in the oncology literature changed over time?

**Findings:**

This cross-sectional study of 13 general oncology/medicine, radiation oncology, and surgical journals found that female authorship of oncology research articles rose from 25.5% in 2002 to 31.7% in 2018, with considerable variation based on author position, article type, and journal type. This rise is less than the increase in female oncology faculty over that time period.

**Meaning:**

While female authorship in oncology research literature has increased over time, it has not kept up with the increase in female oncology faculty.

## Teaser

This cross-sectional study of 13 oncology research journals examines the trends in female authorship from 2002 to 2018, including positioning in author bylines and in comparison with gender distribution in oncology research faculty.

## Introduction

Despite gender parity in medical school participation in recent years,^1,2^ gender disparity remains in the numbers of practicing physicians, with variations by country^3^ and specialty.^4^ Within academic centers in the United States, these discrepancies become more apparent with increasing academic rank,^5^ reflecting “the leaky pipeline” of academic medicine.

Successful academic careers often hinge on the publication of peer-reviewed research. However, many systematic biases can specifically hinder women, such as the possibility of unconscious bias in peer review that may influence how women present their research,^6,7,8^ along with underrepresentation on the editorial boards of major medical journals, which may lead journals to be less likely to prioritize subjects more commonly studied by women or create networks that exclude them.^9,10,11^ Additionally, women are underpaid relative to their male counterparts within academia and receive smaller start-up packages,^12,13,14^ receive less mentorship^15^ and sponsorship,^16^ face overt discrimination and harassment,^17^ and often make personal or professional sacrifices because of traditional gender roles.^18,19^

Although there has been an upward publishing trend in medicine, the proportion of female authors in the medical literature varies greatly by field, authorship position, and journal.^20^ Fields such as obstetrics and gynecology and pediatrics generally have higher rates of female authorship.^21^ Even within these fields, the percentage of female authors depends on authorship position, with the last or senior author position having the smallest representation, and on author rank, with women’s representation being greatest among medical student authors and lowest among authors who are full professors.^20,21,22^ Different journals have varying rates of female first-authorship,^23^ and women are underrepresented as presenters at major medical conferences.^24^

In general, female authorship in oncology has been increasing,^21^ again with differences based on author position and discipline.^25^ The *International Journal of Radiation Oncology, Biology, Physics* (*IJROBP*), the radiation oncology journal with the highest impact factor and the most studied oncology journal, showed an increase in female authorship from 1980 through 2012 that corresponds with the rise in female full-time radiation oncology faculty and residents.^26^

Though previous work has examined trends in female authorship by sampling articles from a few years in a small number of journals, this study examines a more comprehensive data set: the entirety of the Medline-indexed oncology literature in high impact factor medical journals over a 17-year span.

## Methods

### Data Collection

Data for this cross-sectional bibliometric analysis were collected from Medline via Medical Subject Headings (MeSH) term searches built in legacy PubMed. Thirteen clinically oriented journals with high impact factors for their respective fields and whose Medline citations included full first names were individually searched for oncology-specific articles. These journals were the *Journal of the American Medical Association* (*JAMA*), the *Lancet*, the *New England Journal of Medicine*, *JAMA Oncology*, *Lancet Oncology*, *Journal of Clinical Oncology*, *IJROBP*, *Radiotherapy and Oncology*, *Practical Radiation Oncology*, *Annals of Surgical Oncology*, *Journal of Surgical Oncology*, and *Annals of Surgery*. For each journal, separate searches were performed for each of 5 article types: clinical trials, observational studies (excluding case reports), systematic reviews with or without meta-analysis, general reviews, and other articles including letters, correspondences, news, replies, comments, and editorials (see eAppendix in the Supplement for exact search terms used). Analysis was restricted from 2002 through 2018 because author first names were not regularly included in Medline prior to 2002 and many of the included journals did not have all of their articles indexed with assigned MeSH terms for 2019 and later. Full lists of Medline citations for all resulting articles were directly exported from PubMed. No other exclusion criteria were applied.

### Data Categorization

Authors for each extracted article were categorized as a first author, second author, last author, or other author based on their position in the extracted citation. Articles with a sole author had the author designated as a first author only, while those with 2 authors had the authors designated as a first or last author only. Last authorship was used as a surrogate for senior authorship because we were unable to efficiently extract the name of the corresponding author from the numerous articles included in the study. Journals were grouped either into general oncology, radiation oncology, or surgical oncology. Articles were categorized into 3 main groups. Primary articles included clinical trials and observational studies, such as cohort or cross-sectional studies. Secondary articles were reviews, systematic reviews, and meta-analyses. The remaining articles—including letters, correspondences, and replies—were grouped together as other articles. First names were used to infer authors’ genders based on societal naming norms via the third-party service Gender-API.com (Markus Perl). Using their database comprising names from 191 different countries and algorithm, names were assigned as either male or female with an estimated accuracy score ranging from 50 through 100, with 50 signifying complete uncertainty and 100 signifying complete certainty in a name’s gender. This service was used because it outperformed similar name-to-gender platforms in culturally diverse data sets.^27^

### Data Validation and Statistical Analysis

Data were analyzed between April and May 2020. All data analysis and statistical testing was performed in R version 3.6.1 (R Foundation for Statistical Computing) with a 2-tailed significance level prespecified at α = .05. Adjustments for multiple comparisons were not performed. Though the Gender-API.com service has been previously validated,^27^ internal validation was performed. Authors were randomly chosen until 500 were successfully assigned genders manually based on Google searches masked to the algorithmically assigned gender. Accuracy scores were scaled such that 0 and 100 corresponded to absolute certainty of male or female gender, respectively, with the midpoint representing complete uncertainty. Simple logistic regression was performed using the scaled accuracy scores to predict the manually assigned author genders. A receiver operating characteristic curve was generated from the model, and Youden’s index was used to find the optimal cutoff of the accuracy score.

For the entire data set, percentages of female authorship by journal specialty, publication type, and authorship position were tabulated for each year. Comparisons between groups were performed using a Pearson χ^2^ test of independence. The subset of primary journal articles (clinical trials and observational studies) were divided by discipline (general, radiation oncology, or surgical oncology), and multivariable logistic regression was performed with publication year, authorship position, and primary article type, as well as all 2-way and 3-way interactions, as independent variables to predict female authorship. Publication year was entered as years after 2002, and all categorical variables were dummy-coded. The Box-Tidwell test was used to confirm linearity between year and the log-odds of female authorship.

## Results

### Data Summary and Validation

A total of 420 526 authors from 58 368 articles were found over the 17 years spanning 2002 through 2018. Of those authors, 400 945 were assigned a gender based on their name through Gender-API.com. Among the 19 581 authors for whom a gender was not assigned, 15 469 (79.0%) had only a first initial present in their Medline reference. Overall, Gender*-*API performed well, with a concordance index of 0.994 (95% CI, 0.989-0.998) between its scaled accuracy score and manually assigned author genders. The optimal cutoff was found to be at a scaled accuracy of 49, corresponding to correctly identifying 349 of 359 men (97.2%) and 136 of 141 women (96.5%).

The breakdown of the number of authors for whom a gender was assigned and the number of corresponding articles by discipline and publication type is shown in Table 1. In total, 29.5% (95% CI, 29.4%-29.6%) of authors were identified as female, rising from 25.5% (95% CI, 24.7%-26.3%) in 2002 to 31.7% (95% CI, 31.2%-32.3%) in 2018. The most common article type in the general oncology journals were clinical trials, closely followed by observational studies. In contrast, the most common article types in both radiation oncology and surgical oncology journals were observational studies. The percentage of female authors over 3-year spans at the beginning and end of the studied time frame, stratified by publication type and discipline, can be seen in Table 2. General oncology journals had a rise in female authorship from 28.9% (95% CI, 28.3%-29.5%) to 34.5% (95% CI, 34.1%-34.9%). Radiation oncology journals had a rise in female authorship from 23.5% (95% CI, 22.7%-24.3%) to 31.5% (95% CI, 30.9%-32.1%). Surgical oncology journals had a rise in female authorship from 20.4% (95% CI, 19.4%-21.4%) to 28.2% (95% CI, 27.6%-28.8%).

**Table 1.** Total Number of Articles and Authors in Each Journal Type, Categorized by Publication Type

Publication type	No. (%)					
	General		Radiation		Surgical	
	Articles (n = 26 828)	Authors (n = 186 696)	Articles (n = 15 396)	Authors (n = 119 594)	Articles (n = 13 529)	Authors (n = 94 655)
Trial	5353 (20.0)	80 168 (42.9)	2232 (14.5)	21 744 (18.2)	999 (7.4)	9184 (9.7)
Observational	7488 (27.9)	67 125 (36.0)	11 340 (73.7)	90 792 (75.9)	9508 (70.3)	76 610 (80.9)
Systematic review	203 (0.8)	2524 (1.4)	43 (0.3)	322 (0.3)	83 (0.6)	545 (0.6)
Review	2427 (9.0)	10 164 (5.4)	529 (3.4)	3152 (2.6)	907 (6.7)	3479 (3.7)
Other	11 357 (42.3)	26 715 (14.3)	1252 (8.1)	3584 (3.0)	2032 (15.0)	4837 (5.1)

**Table 2.** Percentage of Female Authors Over 3-Year Spans at Beginning and End of Study Period, Stratified by Discipline and Study Type^a^

Publication type	% (95% CI)					
	General		Radiation		Surgical	
	2002-2004	2016-2018	2002-2004	2016-2018	2002-2004	2016-2018
Trial	27.5 (26.5-28.5)	32.1 (31.4-32.8)	24.9 (23.1-26.7)	33.2 (31.9-34.5)	23.3 (20.4-26.2)	29.0 (27.1-30.9)
Observational	32.7 (31.6-33.8)	39.2 (38.4-40.0)	23.7 (22.8-24.6)	31.2 (30.5-31.9)	20.1 (19.0-21.2)	28.3 (27.7-28.9)
Secondary	26.0 (23.4-28.6)	34.3 (32.6-36.0)	18.5 (14.2-22.8)	32.1 (29.4-34.8)	21.3 (17.5-25.1)	32.8 (29.5-36.1)
Other^b^	23.9 (22.4-25.4)	30.5 (29.4-31.6)	13.6 (9.9-17.3)	26.4 (23.1-29.7)	15.8 (11.8-19.8)	23.8 (21.8-25.8)
Total	28.9 (28.3-29.5)	34.5 (34.1-34.9)	23.5 (22.7-24.3)	31.5 (30.9-32.1)	20.4 (19.4-21.4)	28.2 (27.6-28.8)

^a^ Three-year spans were used to help mitigate any year-to-year variation in comparing the beginning and end of this time frame, although presentation in this way blunts the small increase in female authorship within each of the 2 3-year spans.

^b^ Other published works includes letters, correspondences, news, replies, comments, and editorials.

### Primary Literature

**Figure 1.**
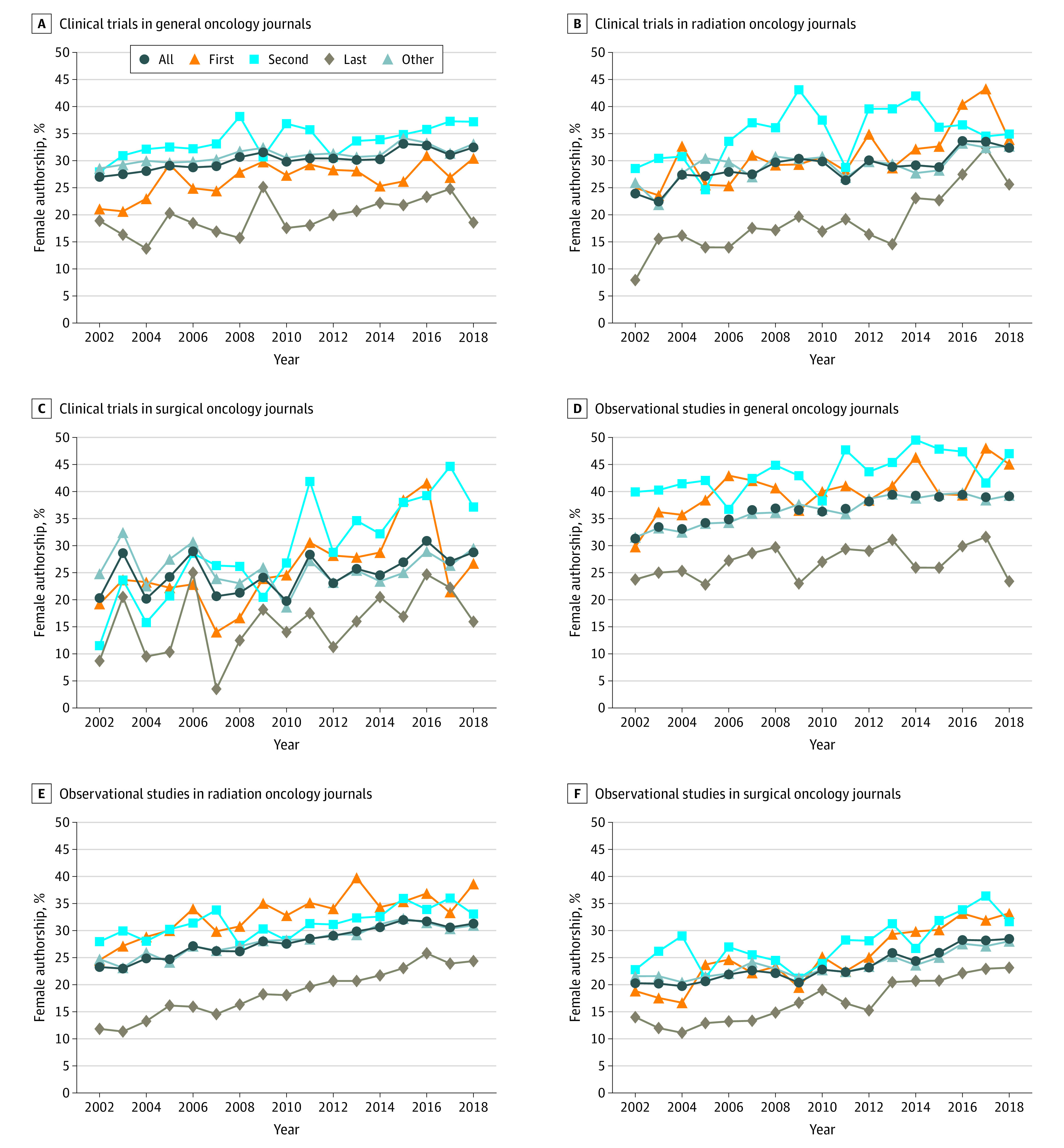
Female Authorship Over Time in Primary Oncology Articles by Subspecialty

With the overall rise in female authorship for oncology-related journal articles, there were notable differences based on the specialty, publication type, and authorship position. The initial focus of this analysis was on primary research articles because of their relatively higher importance for career advancement. Figure 1 shows the percentage of female authors by discipline and authorship position vs year for clinical trials and observational studies. Each subgroup showed increased female authorship with each increasing year and the full results of the corresponding logistic regression models can be found in Table 3.

**Table 3.** Results of Logistic Regression Models for Predictors of Female Authorship of Primary Articles in Each of the 3 Journal Types

Characteristic	Odds ratio (95% CI)		
	General	Radiation	Surgical
Annual overall increase	1.02 (1.01-1.03)	NA	NA
First authors	NA	1.03 (1.02-1.04)	1.06 (1.04-1.07)
Second authors			1.04 (1.03-1.05)
Last authors		1.06 (1.05-1.07)	1.06 (1.04-1.07)
Other authors		1.03 (1.02-1.04)	1.03 (1.02-1.04)
Last author vs First author regardless of primary article type	0.60 (0.52-0.69)	2002: 0.36 (0.31-0.42); 2018: 0.57 (0.53-0.60)	0.61 (0.50-0.73)
Second author vs First author in observational studies	1.18 (1.03-1.35)	1.00 (0.88-1.12)	2002: 1.31 (1.11-1.55); 2018: 0.99 (0.94-1.05)
Second author vs First author in clinical trials		1.39 (1.06-1.82)	
Other author vs First author in observational studies	0.89 (0.80-0.98)	0.82 (0.75-0.91)	2002: 1.18 (1.03-1.35); 2018: 0.74 (0.71-0.77)
Other author vs First author in clinical trials	1.29 (1.13-1.47)	0.90 (0.81-1.00)	
Clinical trial vs observational study			
First authors	0.58 (0.49-0.67)	0.83 (0.67-1.03)	1.09 (0.75-1.56)
Second authors		1.16 (0.94-1.42)	
Last authors		0.83 (0.67-1.03)	
Other authors	0.84 (0.79-0.89)	1.12 (1.03-1.23)	

Each column represents an individual model where multivariable logistic regression was performed with publication year (continuous variable), authorship position (first, second, last, or other author), primary article type (observational study or clinical trial), and all 2-way and 3-way interactions as independent predictors of female authorship. All 3-way interactions were insignificant and excluded from this table. Merged rows within a column (or identical values if rows are not contiguous) reflect variables where interaction terms were insignificant, so the variables of interest share a common odds ratio. For variables with statistically significant interactions with year, calculated odds ratios for the year 2002 and 2018 were included in the table.

Among all articles published in general oncology journals, second authors were more likely to be women than first authors (Odds ratio [OR], 1.18; 95% CI, 1.03-1.35), whereas last authors were less likely to be women than first authors (OR, 0.60; 95% CI, 0.52-0.69). Women were less likely to be authors of clinical trials at each authorship position than authors at that respective position for observational studies (first, second, and last authors: OR, 0.58; 95% CI, 0.49-0.67; other authors: OR, 0.84; 95% CI, 0.79-0.89). For observational studies, other authors were less likely to women as compared with first authors (OR, 0.89; 95% CI, 0.80-0.98), though for trials, other authors were more likely to be women than first authors (OR, 1.29; 95% CI, 1.13-1.47).

For articles published in radiation oncology journals, last authors were less likely to be women than first authors, though the relative gap appeared to narrow with time (2002: OR, 0.36; 95% CI, 0.31-0.42 vs 2018: OR, 0.57; 95% CI, 0.53-0.60). For observational studies, there was no difference in the odds of female authorship between second and first authors, though other authors were less likely to be women than first authors (OR, 0.82; 95% CI, 0.75-0.91). For trials, second authors were more likely to be women then first authors (OR, 1.39; 95% CI, 1.06-1.82), although there was no difference in the odds of other authors being women as compared with first authors (OR, 1.11; 95% CI, 0.90-1.37).

In surgical oncology journals, last authors were again less likely to be women than first authors (OR, 0.61; 95% CI, 0.50-0.73). Second authors were more likely to be women than first authors early in the timeframe, although the discrepancy disappeared over time (2002: OR 1.31, 95% CI, 1.11-1.55 in 2002 vs 2018: OR, 0.99; 95% CI, 0.94-1.05). Similarly, other authors were more likely to be women than first authors early on, though the discrepancy reversed over time (2002: OR, 1.18; 95% CI, 1.03-1.35 vs 2018: OR, 0.74; 95% CI, 0.71-0.77). No differences in female authorship were seen between trials and observational studies.

### Secondary Literature and Other Articles

**Figure 2.**
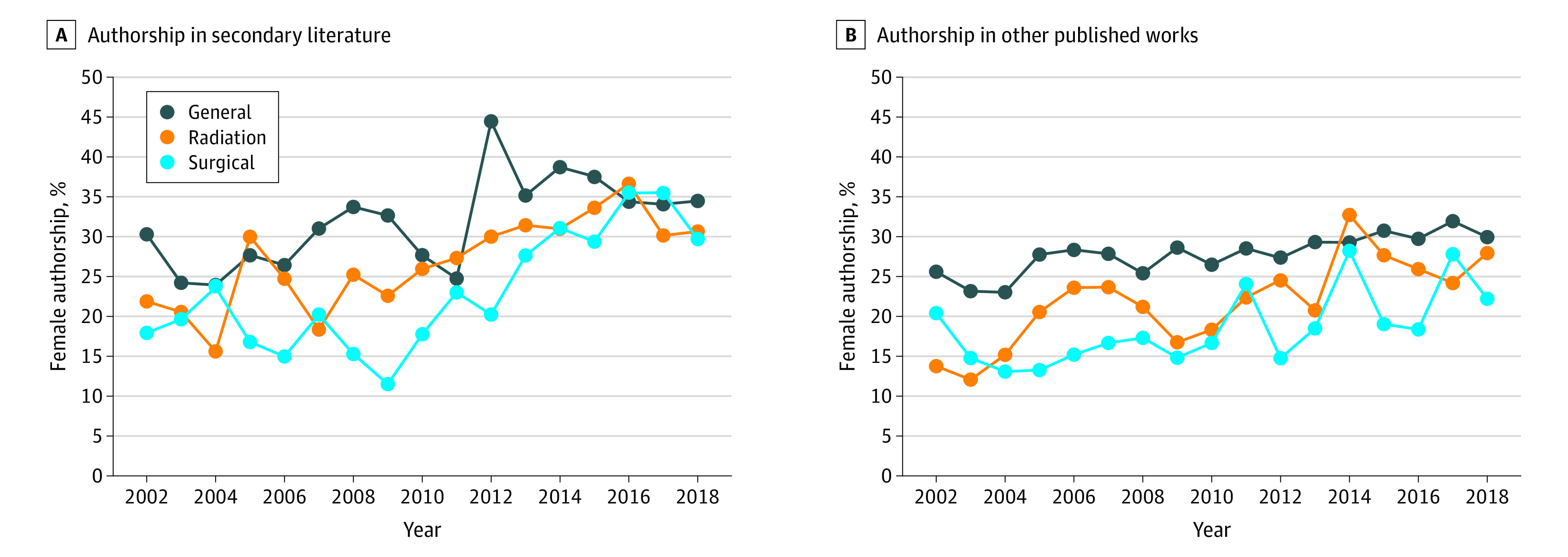
Female Authorship Over Time in Secondary and All Other Oncology Articles by Subspecialty Other published works include letters, correspondences, news, replies, comments, and editorials.

Figure 2 shows the overall percentage of female authors for secondary literature and other published articles for each discipline. The percentage of authors who were female at the beginning and end of the studied time span for these article types stratified by discipline can be seen in Table 2. Between 2016 and 2018, the percentage of female secondary literature authors was comparable with that in primary literature for general oncology journals (OR, 1.04; 95% CI, 0.96-1.13) and radiation oncology journals (OR, 0.98; 95% CI, 0.86-1.11). For surgical oncology journals, the percentage of female authors for secondary literature during that recent time span was greater than that for primary literature (OR, 1.25; 95% CI, 1.07-1.45). Female authorship in all other articles was considerably lower than that in primary articles for general oncology (OR, 1.24; 95% CI, 1.17-1.31), radiation oncology (OR, 1.29; 95% CI, 1.08-1.53), and surgical oncology journals (OR, 1.27; 95% CI, 1.14-1.43) from 2016 to 2018.

## Discussion

To our knowledge, this article presents the largest study of female authorship in oncology literature to date. It demonstrates that female authorship has increased over the past 17 years. To put these numbers in context, it is useful to examine the corresponding change in the proportion of female trainees and faculty members over this time span. These estimates come from Association of American Medical Colleges data,^4^ as well as 2 analyses by Chowdhary et al^28^ and Ahmed et al.^29^ The percentage of female hematology oncology trainees has increased from 38.5% in 2002 to 42.9% in 2017, although most years past 2010 have seen more than 45% female trainees. The percentage of female hematology-oncology faculty has also seen an increase from 28.7% in 2002 to 37.1% in 2018, with a peak of 38.8% in 2015. Overall, despite the approximately 9% to 10% increase in female hematology oncology faculty and the relative persistence of more than 45% female hematology-oncology trainees annually, this study reveals only about a 5.6% increase to 34.5% in female authorship of general oncology journal articles.

Unfortunately, the percentage of female radiation oncology trainees has remained relatively stagnant from 30.0% in 2002 to 29.4% in 2017, with a peak of 35% in 2007. The percentage of female radiation oncology faculty has increased from 23.8% in 2002 to 30.7% in 2018. This increase in female faculty is roughly in line with the increase in female authorship in radiation oncology journals found in this study, which rose from approximately 23.5% to 31.5%. The percentage of female general surgery trainees has increased from 30.8% in 2007 to 40.1% in 2017, and it is estimated that as of 2018, 38.8% of surgical oncology faculty are women. The percentage of female authors in surgical oncology journals in this study has lagged behind these numbers at only approximately 28.2% (95% CI, 27.6%-28.8%).

Dalal et al^25^ described similar upward trends in the oncology literature. They analyzed all original articles, letters, notes, editorials, reviews, and proceedings in 5 major oncology journals at 4 different time points between 1990 and 2017. Among first authors in 2017, they found approximately 31% in the *Annals of Surgical Oncology* were women, approximately 34% in the *IJROBP* and *Journal of Clinical Oncology*, approximately 37% in *JAMA Oncology*, and approximately 45% in *Cancer*. For each journal that year, the percentage of female senior authors was nearly 10% lower than that for first authors. Ahmed et al^26^ examined trends in female authorship in the *IJROBP* through 2012. For all original articles in 2012, they found 29.7% of first authors and 22.6% of senior authors were women. The results of our study for those 2 years (Figures 1 and 2) are similar to these previous works, with small differences likely due to the averaging of multiple journals in this analysis.

Across all disciplines, last authors of primary articles were less likely to be women than first authors, suggesting fewer women in senior positions. This discrepancy was seen in other studies^20,21,25,26^ and is not surprising given the surrounding academic framework. Chowdhary et al^28^ demonstrated that among all accredited training programs in the US in early 2020, 37.1% of medical oncology faculty, 30.7% of radiation oncology faculty, and 38.8% of surgical oncology faculty were women. There were even fewer women in chair positions, at 21.7%, 11.7%, and 3.8%, respectively. Other studies document the differential challenges that affect men and women serving as radiation oncology chairs.^30,31^ A separate study by Hofstädter-Thalmann et al^32^ in 2018 found women were poorly represented among board members of international cancer societies, including 7.1% for American Society for Radiation Oncology, 14.3% for the European Society for Medical Oncology, 25.0% for the American Society for Clinical Oncology, and 28.6% for the European Organisation for Research and Treatment of Cancer. Moreover, women were poorly represented as invited speakers at these societies’ congresses. The persistent dearth of women among last authors likely reflects this underrepresentation higher in academia as well as previously mentioned systemic biases.

One interesting and novel finding of the current study is the discrepancy in female authorship in general oncology journals between clinical trials and observational studies (Table 2 and Figure 1). In general, female authorship in observational studies in general oncology journals is higher than in any primary article type for any other journal type. This gap was not seen for radiation oncology or surgical oncology journals, and to the authors’ knowledge it has not been noted previously. Ludmir et al^33^ analyzed 598 phase 3 oncologic randomized control trials and noted that industry-funded studies had lower rates of female first authorship while cooperative group trials had higher rates. Additionally, they noted that breast and head and neck trials tended to have higher rates of female first authorship vs other sites. The gap seen between clinical trials and observational studies in general oncology journals is likely to be multifactorial. Understanding why women might be less well represented in authoring those studies merits further investigation, particularly because clinical trials in general oncology may be especially influential. It should be noted that the present study did not stratify articles by topic, funding source, phase, or presence of randomization.

### Limitations

This study had several limitations. While the goal of this study was to describe the advancement of women in oncology, the use of author names, which are often assigned at birth based on biological sex, are an imperfect representation of the gender identity of an individual author. Additionally, this analysis uses a simplistic construct of gender as binary, when in reality gender exists on a spectrum. Another limitation of this study comes from any systemic biases arising from Medline indexing, MeSH term assignment (or lack of assignment), and gender assignment through Gender-API.com. However, given the likelihood that this sample encompasses most of the oncologic literature published in these journals, any such biases are likely minimal. These findings represent journals with relatively high impact factors for their fields and may not be generalizable to the entire fields. While general oncology journals tend to have mostly medical oncology articles, they do contain some radiation and surgical oncology articles, reducing the specificity of this group to medical oncology. Additionally, all authors were included in this analysis regardless of degree, cofirst or cosenior authors were not addressed, and last author is not a perfect surrogate for senior author.

## Conclusions

Overall, this work samples most of the oncologic literature in 13 well-regarded journals to analyze trends in female authorship over 17 years. While the percentage of women among authors in major oncology journals has increased, women remain in the minority, with the proportion of women among authors in surgical and general oncology journals lagging behind the proportions serving as surgical and hematology-oncology faculty.

## Notes

Supplement. **eAppendix.** Search Terms Used in PubMed for Data Acquisition
