# Supplementary material for: Trends in Female Authorship in Major Journals of 3 Oncology Disciplines, 2002-2018
Source: JAMA Netw Open. 2021 Apr 6;4(4):e212252. doi: 10.1001/jamanetworkopen.2021.2252 (PMC8025110; doi:10.1001/jamanetworkopen.2021.2252)
Supplement: Supplement. — eAppendix. Search Terms Used in PubMed for Data Acquisition [file jamanetwopen-e212252-s001.pdf]

Supplemental Online Content

Yalamanchali A, Zhang ES, Jagsi R. Trends in female authorship in major journals of 3 oncology disciplines, 2002-2018. *JAMA Netw Open*. 2021;4(4):e212252. doi:10.1001/jamanetworkopen.2021.2252

eAppendix. Search Terms Used in PubMed for Data Acquisition

This supplemental material has been provided by the authors to give readers additional information about their work.

© 2021 Yalamanchali A et al. *JAMA Network Open.*

eAppendix. Search terms used in PubMed for data acquisition.

“Journal Title” is replaced by one of the thirteen journals of interest. Separate search terms were used for clinical trials, observational studies, systematic reviews, general reviews, and all other articles.

"Journal Title"[Journal] AND (Clinical Trial[Publication Type]) AND (neoplasm[MeSH Terms] OR antineoplastic protocols[MeSH Terms] OR cancer pain[MeSH Terms] OR hematopoietic stem cell transplantation[MeSH Terms] OR neoplasm staging[MeSH Terms]) NOT cysts[MeSH Terms] NOT letter[Publication Type] NOT editorial[Publication Type] NOT review[Publication Type] NOT systematic review[Publication Type] NOT meta-analysis[Publication Type]

"Journal Title"[Journal] AND (neoplasm[MeSH Terms] OR antineoplastic protocols[MeSH Terms] OR cancer pain[MeSH Terms] OR hematopoietic stem cell transplantation[MeSH Terms] OR neoplasm staging[MeSH Terms]) NOT cysts[MeSH Terms] NOT letter[Publication Type] NOT editorial[Publication Type] NOT review[Publication Type] NOT systematic review[Publication Type] NOT meta-analysis[Publication Type] NOT clinical trial[Publication Type] NOT case reports[Publication Type] NOT comment[Publication Type] NOT news [Publication Type]

"Journal Title"[Journal] AND (systematic review[Publication Type] OR meta-analysis[Publication Type]) AND (neoplasm[MeSH Terms] OR antineoplastic protocols[MeSH Terms] OR cancer pain[MeSH Terms] OR hematopoietic stem cell transplantation[MeSH Terms] OR neoplasm staging[MeSH Terms]) NOT cysts[MeSH Terms] NOT letter[Publication Type] NOT editorial[Publication Type] NOT review[Publication Type] NOT clinical trial[Publication Type]

"Journal Title"[Journal] AND (review [Publication Type]) AND (neoplasm[MeSH Terms] OR antineoplastic protocols[MeSH Terms] OR cancer pain[MeSH Terms] OR hematopoietic stem cell transplantation[MeSH Terms] OR neoplasm staging[MeSH Terms]) NOT cysts[MeSH Terms] NOT letter[Publication Type] NOT editorial[Publication Type] NOT systematic review[Publication Type] NOT meta-analysis[Publication Type] NOT clinical trial[Publication Type]

"Journal Title"[Journal] AND (letter[Publication Type] OR editorial [Publication Type] or comment[Publication Type] or news[Publication Type]) AND (neoplasm[MeSH Terms] OR antineoplastic protocols[MeSH Terms] OR cancer pain[MeSH Terms] OR hematopoietic stem cell transplantation[MeSH Terms] OR neoplasm staging[MeSH Terms]) NOT cysts[MeSH Terms] NOT review[Publication Type] NOT systematic review[Publication Type] NOT meta-analysis[Publication Type] NOT clinical trial[Publication Type]

© 2021 Yalamanchali A et al. *JAMA Network Open.*
